# Human umbilical cord mesenchymal stromal cells and their small extracellular vesicles modulate host responses to titanium-coated polypropylene mesh in the vaginal wall of rats

**DOI:** 10.3389/fbioe.2026.1985008

**Published:** 2026-10-21

**Authors:** Fangfang Ai, Chunbin Wang, Gang Wang, Xi Wen, Zhiguo Chen, Shijun Wang

**Affiliations:** 1 Department of Obstetrics and Gynecology, Xuanwu Hospital, Capital Medical University, Beijing, China; 2 Cell Therapy Center, Beijing Municipal Geriatric Medical Research Center, Xuanwu Hospital, Capital Medical University, Beijing, China; 3 National Clinical Research Center for Geriatric Diseases, and Key Laboratory of Neurodegenerative Diseases, Ministry of Education, Beijing, China; 4 Department of Central Laboratory, The First Affiliated Hospital of Lishui University, Lishui People's Hospital, Lishui, Zhejiang, China

**Keywords:** extracellular matrix, mesenchymal stromal cells, polypropylene, small extracellular vesicles, surgical mesh

## Abstract

**Objective:**

To assess whether human umbilical cord mesenchymal stromal cells (HUCMSCs) or HUCMSC-derived small extracellular vesicles (HUCMSC-sEVs) modify tissue responses to titanium-coated polypropylene mesh in the rat vaginal wall.

**Methods:**

Fifty-four ovariectomized female Sprague–Dawley rats were randomly assigned to saline-control, HUCMSC-sEV, or HUCMSC groups (18 each). After mesh implantation, animals received one 0.3-mL local injection. Independent cohorts were assessed at 1, 4, and 12 weeks. Hematoxylin and eosin staining evaluated inflammatory cell infiltration, neovascularization, and fibrous encapsulation; picrosirius red staining assessed collagen composition and the COL I/COL III ratio; and quantitative real-time polymerase chain reaction measured inflammation- and matrix-remodeling-related genes. The animal was the experimental unit, and false discovery rate adjustment was applied to multiple comparisons.

**Results:**

At week 1, inflammatory cell infiltration was lower in the HUCMSC-sEV group than in the saline-control group, while interleukin-10, interleukin-12, and tumor necrosis factor-α mRNA expression was lower in both intervention groups. At week 4, both interventions reduced fibrous encapsulation and increased matrix metalloproteinase-2 and -9 mRNA expression. At week 12, the type I/type III collagen ratio was lower in both intervention groups, matrix metalloproteinase-2 and -9 mRNA expression was higher in both intervention groups, and type III collagen gene expression was higher in the HUCMSC group.

**Conclusion:**

HUCMSCs and HUCMSC-sEVs were associated with changes in early inflammatory cell infiltration and cytokine-transcript expression, fibrous encapsulation, and later collagen- and matrix-related measures after mesh implantation. The partially overlapping response patterns observed between HUCMSC-sEVs and HUCMSCs support further investigation of HUCMSC-sEVs as a cell-free approach to modulating mesh-associated host responses.

## Introduction

Pelvic organ prolapse is a common pelvic floor disorder that can substantially impair urinary, bowel, sexual, and overall quality-of-life outcomes (Barber, 2016; Collins and Lewicky-Gaupp, 2022; Ackenbom, 2026). Surgery remains an important treatment for symptomatic or advanced disease, and polypropylene mesh has been used to provide durable mechanical support during pelvic floor reconstruction (Huang et al., 2021; Zhao et al., 2026). However, as a permanent foreign material, polypropylene mesh may provoke persistent inflammation and fibrosis, contributing to exposure, erosion, contraction, pain, and infection (Whooley et al., 2020; Lin et al., 2022; Seifalian et al., 2023). Improving mesh–host tissue interactions while limiting excessive foreign-body responses therefore remains an important goal in pelvic floor reconstructive surgery.

The host response to mesh implantation involves inflammation, angiogenesis, collagen deposition, and extracellular matrix remodeling. Although controlled inflammation and fibrous tissue formation support mesh fixation and tissue repair, excessive or prolonged responses may disturb collagen homeostasis and promote dense fibrous encapsulation (Sousa et al., 2022; Sleiman et al., 2024; Liu et al., 2025). Type I collagen contributes primarily to tensile strength, whereas type III collagen is associated with a more compliant reparative matrix. Matrix metalloproteinases (MMPs) and their tissue inhibitors further regulate extracellular matrix degradation and turnover (Chi et al., 2022; Gandolfi et al., 2024). Evaluating inflammation, fibrous encapsulation, collagen composition, and matrix-related gene expression may therefore provide a more comprehensive assessment of the host tissue response after mesh implantation.

Human umbilical cord mesenchymal stromal cells (HUCMSCs) have immunomodulatory and tissue-reparative properties and can regulate angiogenesis and extracellular matrix metabolism through paracrine signaling (Lim and Phan, 2014; Ding et al., 2015; Zhang et al., 2022). Previous animal studies have suggested that local HUCMSC administration may modify inflammation and remodeling around polypropylene mesh (Deng et al., 2020; Mao et al., 2021). However, living-cell therapy presents practical challenges related to cell viability, tissue retention, storage, transportation, batch consistency, and quality control. HUCMSC-derived extracellular vesicles (EVs) carry biologically active proteins, lipids, messenger RNAs, and microRNAs and may retain part of the regulatory activity of their parent cells without the proliferative and differentiation-related concerns associated with living-cell administration (Zhang et al., 2021; Krylova and Feng, 2023; Wang et al., 2023; Dehghani et al., 2024). Emerging evidence supports the ability of MSC-derived EVs to modulate inflammation, angiogenesis, and collagen metabolism (Li et al., 2024; Liu and Liu, 2024).

The effects of HUCMSC-derived small extracellular vesicles (HUCMSC-sEVs) on inflammation, fibrous encapsulation, and collagen remodeling after vaginal titanium-coated polypropylene mesh implantation have not been systematically characterized, and direct comparisons with HUCMSCs in the same experimental model remain limited. In this study, we locally administered HUCMSCs, HUCMSC-sEVs, or saline in a rat posterior vaginal wall titanium-coated polypropylene mesh implantation model and evaluated tissue responses at 1, 4, and 12 weeks. We aimed to determine how the two interventions influence inflammation- and extracellular-matrix-related host-response features at different post-implantation time points and to assess whether HUCMSC-sEVs warrant further investigation as a cell-free approach to modulating mesh-associated host responses.

## Materials and methods

### Study design and ethics

A posterior vaginal wall titanium-coated polypropylene mesh implantation model was established in female Sprague–Dawley (SD) rats to compare the effects of local administration of saline, human umbilical cord mesenchymal stromal cells (HUCMSCs), and HUCMSC-derived small extracellular vesicles (HUCMSC-sEVs). Independent terminal cohorts were evaluated at 1, 4, and 12 weeks after surgery. Each animal contributed to only one time point, and no longitudinal repeated measurements were performed.

The protocol was approved by the Animal Ethics Committee of Xuanwu Hospital, Capital Medical University (approval No. XW-20230804-1). All procedures followed institutional guidelines for the care and use of laboratory animals.

### Source and culture of HUCMSCs

Human umbilical cord mesenchymal stromal cells (HUCMSCs) used in all experiments were commercially obtained from Sangon Biotech (Shanghai) Co., Ltd. (Shanghai, China; product No. E601115). No human umbilical cord tissue was collected, processed, or accessed directly by the authors. Cells were cultured under standard conditions at 37 °C with 5% CO_2_ and were used at passages 3–6. Archived study records did not contain quantitative flow-cytometry data or complete trilineage-differentiation results for the commercial HUCMSC product; therefore, the cells were not independently verified in this study against the complete established MSC characterization criteria.

### Isolation and characterization of HUCMSC-derived small extracellular vesicles

At 80%–90% confluence, the commercially obtained HUCMSCs were washed twice and cultured for 48 h in serum-free DMEM/F-12. Cell viability remained above 92% after 48 h of serum-free conditioning before conditioned-medium collection. The conditioned medium was centrifuged at 500 × g for 10 min and 2,000 × g for 20 min at 4 °C, followed by filtration through a 0.22-μm membrane. The filtrate was concentrated to approximately 200 μL in a 100-kDa ultrafiltration unit at 4,000 × g for 40 min at 4 °C. After dilution with phosphate-buffered saline (PBS), the concentrate was ultracentrifuged at 100,000 × g for 90 min at 4 °C. The pellet was resuspended and washed by a second ultracentrifugation under the same conditions, then aliquoted and stored at −80 °C.

Vesicle morphology was evaluated by transmission electron microscopy (TEM; Hitachi). Briefly, 10 μL of the sEV suspension was applied to a copper grid and incubated for 10 min at room temperature. After washing with sterile distilled water, the grid was negatively stained with 10 μL of 2% uranyl acetate for 1 min, excess liquid was removed, and the grid was air-dried for 2 min before imaging at 80 kV. Particle size distribution and concentration were determined by nanoparticle tracking analysis using a ZetaView PMX 110 instrument (Particle Metrix, Germany). Samples were diluted in PBS to approximately 1 × 10^7^ to 1 × 10^9^ particles/mL before measurement. Measurements were acquired across 11 positions for four cycles using ZetaView software version 8.04.02 SP2. The analysis settings included a maximum area of 1,000, a minimum area of 5, and a minimum brightness of 20. Western blotting was used to assess the EV-associated proteins CD9, CD63, HSP70, and TSG101, with Calnexin assessed as a non-EV-associated marker. The available NTA measurements were obtained from samples derived from two independently generated sEV production batches and showed mean particle diameters of approximately 129–134 nm and particle concentrations of 1.4 × 10^10^ to 2.3 × 10^10^ particles/mL across the characterized samples. These NTA measurements reflected characterization samples obtained before final dose preparation; the suspensions used for animal administration were subsequently concentrated to 1 × 10^11^ particles/mL before injection. Representative TEM, NTA, and Western blot results are shown in Supplementary Figure S7.

Because the specific endosomal biogenesis of the isolated vesicles was not directly demonstrated, the preparation is referred to throughout this manuscript as HUCMSC-derived small extracellular vesicles (HUCMSC-sEVs), in accordance with MISEV2023 recommendations (Welsh et al., 2024).

### Preparation of the titanium-coated polypropylene mesh

TiLOOP® Mesh light titanium-coated polypropylene mesh (15 cm × 15 cm; REF 6000487; pfm medical titanium gmbh, Nürnberg, Germany) was cut into 0.6 cm × 0.8 cm pieces, immersed in 75% ethanol for 10 min, and exposed to ultraviolet light for at least 4 h before implantation.

### Animal model, group allocation, and interventions

Fifty-four female Sprague–Dawley rats aged 6–8 weeks and weighing 180–220 g underwent a 1-week acclimatization period before experimental procedures. Animals were housed two per cage under specific pathogen-free conditions at approximately 22 °C and 55% relative humidity with a 12-h light/12-h dark cycle and had free access to food and water. Bilateral ovariectomy was performed to establish an estrogen-depleted background relevant to the postmenopausal context in which pelvic organ prolapse commonly occurs. Previous studies in adult female rats showed that regular estrous cycling ceases within 1 week after ovariectomy and that plasma estradiol is markedly reduced by day 7 (Li et al., 2014; Mosquera et al., 2015). An approximately 1-week post-ovariectomy interval has also been used in previous vaginal tissue and mesh-implantation models (Jelinsky et al., 2008; Deng et al., 2020). Accordingly, mesh implantation was performed 1 week after ovariectomy to establish an early estrogen-depleted background and to more closely approximate the vaginal tissue environment relevant to postmenopausal pelvic organ prolapse.

Before group assignment, the 54 rats were individually numbered. A random-number table was used to generate the allocation sequence, and the resulting random numbers were mapped, according to a prespecified rule, to three group codes (A, B, and C) corresponding to the saline-control, HUCMSC-sEV, and HUCMSC groups. Animals were assigned sequentially according to this sequence until 18 animals had been allocated to each group, resulting in a 1:1:1 allocation ratio.

Six animals per group were scheduled for terminal tissue collection at weeks 1, 4, and 12. No formal *a priori* sample-size or power calculation was performed. The planned sample size of six animals per group at each time point was informed by comparable preclinical rodent studies of vaginal mesh-host responses that used six animals per group at individual assessment time points (Lo et al., 2016; Guo et al., 2024).

Rats were anesthetized by intraperitoneal injection of 2% pentobarbital sodium at 30 mg/kg and placed in the supine position. A transverse incision was made at the posterior vaginal introitus, and a submucosal pocket was created by blunt dissection. The mesh was placed flat within the pocket, the incision was sutured, and a single injection was administered into the posterior vaginal wall.

The saline-control group received 0.3 mL of 0.9% sodium chloride solution. The HUCMSC group received 0.3 mL of an HUCMSC suspension at 3 × 10^6^ cells/mL, corresponding to 9 × 10^5^ cells per animal. The HUCMSC-sEV group received 0.3 mL of an HUCMSC-sEV suspension at 1 × 10^11^ particles/mL, corresponding to 3 × 10^10^ particles per animal. The total injection volume was 0.3 mL in all groups. The intervention doses were selected with reference to previously reported preclinical dosing ranges. Local vaginal-wall administration of HUCMSCs at doses on the order of 10^6^ cells per animal has been used in ovariectomized rat models (Mao et al., 2021), whereas particle-based EV doses in the 10^10^-particle range have been evaluated in rat studies, including MSC-EV doses up to 3 × 10^10^ particles per animal (Son et al., 2023) and local administration of approximately 1 × 10^10^ EV particles per treatment (Forteza-Genestra et al., 2024).

The HUCMSC and HUCMSC-sEV doses were selected as intervention-specific working doses and were not intended to represent biologically equivalent doses. HUCMSC dosing was defined by cell number, whereas HUCMSC-sEV dosing was normalized by particle number; no validated conversion factor exists between these fundamentally different dosing units. Both interventions were evaluated in the same animal model, administered locally at the same anatomical site and in the same total injection volume, and assessed against a common saline control using the same prespecified histological and molecular outcomes and follow-up time points. This design permitted comparison of whether the two interventions were associated with overlapping or distinct tissue-response patterns under their respective dosing conditions, but not comparison of dose-normalized potency or assessment of therapeutic equivalence or noninferiority. The experimental workflow is shown in Figure 1.

**FIGURE 1:**
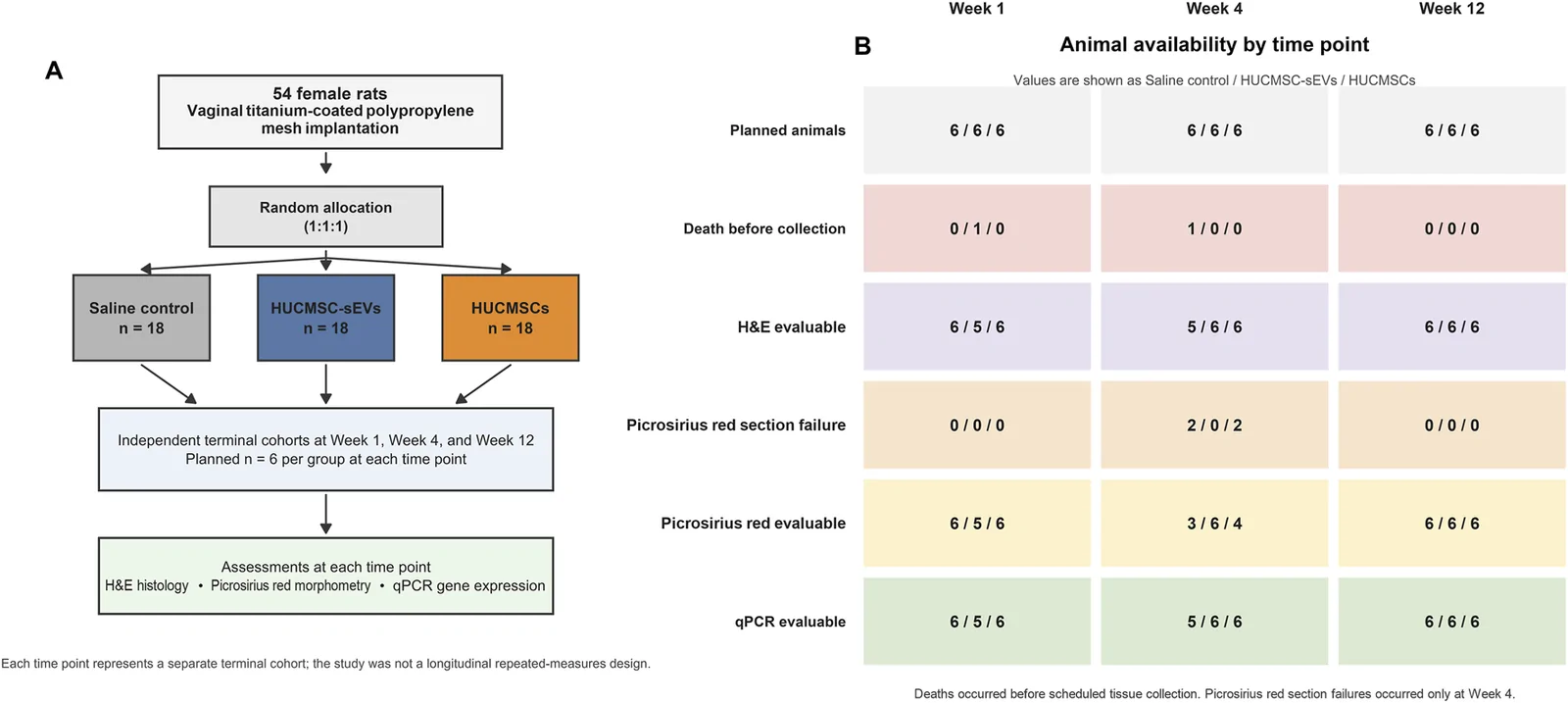
Experimental design, group allocation, and animal availability for histological and qPCR analyses. **(A)** Fifty-four female Sprague–Dawley rats underwent vaginal mesh implantation and were randomly allocated in a 1:1:1 ratio to the saline-control group, HUCMSC-derived small extracellular vesicle (HUCMSC-sEV) group, or human umbilical cord mesenchymal stromal cell (HUCMSC) group, with 18 animals per group. Animals assigned to weeks 1, 4, and 12 constituted independent terminal cohorts, with six animals planned per group at each time point. H&E histology, picrosirius red morphometry, and quantitative real-time polymerase chain reaction (qPCR) were performed on tissue collected at the corresponding terminal time point. Each animal contributed data to only one time point; therefore, the study did not use a longitudinal repeated-measures design. **(B)** Planned and evaluable sample sizes at weeks 1, 4, and 12. Values are presented in the order saline-control group/HUCMSC-sEV group/HUCMSC group. One animal in the HUCMSC-sEV group died before the scheduled week-1 tissue collection, and one animal in the saline-control group died before the scheduled week-4 tissue collection; neither animal contributed evaluable tissue data. Consequently, 52 animals were evaluable for H&E histology and qPCR. At week 4, picrosirius red sections from two animals in the saline-control group and two animals in the HUCMSC group were unavailable because of technical failure during histological section preparation, leaving 48 animals evaluable for picrosirius red morphometry. Missing data were not imputed.

### Animal monitoring and terminal procedures

Rats were anesthetized with 2% pentobarbital sodium for the surgical procedures. No additional postoperative analgesic medication was administered. During postoperative follow-up, animals were monitored for general activity, food intake, wound healing, evidence of bleeding, and body weight. At the scheduled terminal time points of weeks 1, 4, and 12, animals were anesthetized with 2% pentobarbital sodium. Thoracotomy was then performed under anesthesia, and the heart was incised until cardiac activity ceased, after which the vaginal mesh–tissue complex was collected for subsequent analyses.

### Tissue collection and sample sizes

Vaginal mesh–tissue complexes were collected at 1, 4, and 12 weeks after implantation for hematoxylin and eosin (H&E) staining, picrosirius red staining, and quantitative real-time polymerase chain reaction (qPCR). Evaluable sample sizes for each analysis are shown in Figure 1 and Supplementary Table S7. Missing data resulting from animal death or unsuccessful histological section preparation were not imputed.

### H&E staining and histological assessment

Samples were fixed in 10% neutral buffered formalin for at least 24 h, dehydrated through a graded ethanol series, embedded in paraffin, sectioned at approximately 4 μm, and stained with H&E.

One H&E-stained section per animal was evaluated. For each section, two observers who were blinded to treatment allocation independently examined multiple microscopic fields surrounding the implanted mesh and assigned one overall semiquantitative score for each histological feature based on the collective assessment of these fields. Inflammatory cell infiltration, neovascularization, and fibroblast proliferation were graded on a 0–4 ordinal scale (0, absent; 1, minimal; 2, mild; 3, moderate; and 4, severe). Fibroblast proliferation served as a semiquantitative surrogate for the fibrous encapsulation response and is therefore reported throughout the manuscript as the fibrous encapsulation score. The mean of the two observer scores was used as the animal-level value, with the animal serving as the statistical unit.

Interobserver agreement was assessed using linearly weighted Cohen’s *κ*, exact agreement, and agreement within one point. The 95% confidence interval for *κ* was estimated using 5,000 bootstrap resamples, with the animal as the resampling unit.

### Picrosirius red staining and collagen quantification

Picrosirius red-stained sections were examined under polarized light microscopy under standardized image-acquisition conditions. Six non-overlapping microscopic fields surrounding the implanted mesh were acquired from each section and analyzed using Image-Pro Plus 6.0. Under polarized light, collagen fibers exhibited mixed birefringence ranging from green through yellow to red. For morphometric analysis, red- and yellow-birefringent fibers were operationally classified as type I collagen-associated fibers, whereas green-birefringent fibers were operationally classified as type III collagen-associated fibers. Color segmentation was performed using fixed thresholds that were applied consistently to all images. For each field, the area fraction of each collagen category was calculated as the corresponding color-positive area divided by the total image area and reported as an area fraction. Animal-level COL I- and COL III-positive area fractions and the primary COL I/COL III ratio were calculated as the arithmetic means of their respective six field-level values, with the animal serving as the experimental unit. As a sensitivity analysis, an alternative ratio was calculated by dividing the animal-level mean COL I-positive area fraction by the corresponding mean COL III-positive area fraction.

### Quantitative real-time polymerase chain reaction

Total RNA was extracted from vaginal mesh–tissue complexes using the TRIzon method. One microgram of RNA was reverse-transcribed in a total volume of 10 μL at 37 °C for 15 min and 85 °C for 5 s, and the resulting complementary DNA was diluted 1:10.

Amplification was performed using SYBR Green chemistry on an ABI 7500-series real-time polymerase chain reaction system. The cycling protocol consisted of 95 °C for 30 s, followed by 40 cycles of 95 °C for 5 s and 60 °C for 30 s, with melting-curve analysis after amplification.

Three technical replicate wells and three negative-control wells were included for each sample–gene combination. After quality control, the mean cycle threshold (Ct) value of the technical replicates was used for analysis; technical replicates were not treated as independent experimental units. The genes analyzed were COL1, COL3, IL-10, IL-12, MMP2, MMP9, TGF-β, TIMP1, TNF-α, and VEGF, with ACTIN (β-actin) as the reference assay. Primer sequences and reaction conditions are provided in Supplementary Table S8.

ΔCt was calculated as the target-gene Ct minus the ACTIN reference-assay Ct. For each gene and time point, the mean ΔCt of the saline-control group served as the calibrator, and ΔΔCt was calculated as the sample ΔCt minus this mean. Relative expression was calculated using the 2^−ΔΔCt^ method, with the animal as the experimental unit.

### Statistical analysis

The 1-, 4-, and 12-week cohorts were independent, and between-group comparisons were performed separately at each time point. All tests were two-sided, and missing data were not imputed.

H&E scores and picrosirius red outcomes are presented as medians and interquartile ranges. For each assay, three outcomes across three time points yielded nine prespecified omnibus comparisons assessed using the Kruskal–Wallis test. The nine *P* values within each assay were adjusted using the Benjamini–Hochberg false discovery rate (FDR) procedure and reported as *q* values. Dunn pairwise statistics were calculated for completeness, whereas formal *post hoc* interpretation was restricted to outcomes with an omnibus *q* < 0.05; Holm correction was applied within each outcome–time-point combination. Exact permutation tests were used as sensitivity analyses for the primary findings, and the alternative COL I/COL III ratio provided an additional sensitivity analysis for the picrosirius red data.

Primary qPCR analyses were performed on the ΔCt scale. Overall group differences for each gene and time point were assessed using Welch’s one-way analysis of variance, and pairwise comparisons were performed using Welch’s *t*-test. The 60 prespecified intervention-versus-control comparisons were jointly adjusted using the Benjamini–Hochberg procedure. The 30 comparisons between the HUCMSC-sEV and HUCMSC groups were considered exploratory and underwent a separate FDR adjustment. Nonparametric sensitivity analyses were conducted using the Kruskal–Wallis test and exact Mann–Whitney *U* tests.

For FDR-adjusted analyses, *q* < 0.05 was considered statistically significant; for Holm-adjusted pairwise comparisons, an adjusted *P* value < 0.05 was considered statistically significant. Complete results are provided in Supplementary Tables S1–S6 and S9A–S9E. All analyses were performed using R software, version 4.6.0 (R Foundation for Statistical Computing, Vienna, Austria).

## Results

### Animal outcomes and evaluable samples

A total of 54 rats were included, with 18 animals per group. One animal in the HUCMSC-sEV group died before the scheduled week-1 tissue collection, shortly after induction of anesthesia. Limited abdominal examination performed immediately afterward did not identify an obvious site of internal bleeding. Because no formal necropsy was performed, the exact cause of death could not be established; an anesthesia-related complication was considered the most likely explanation based on the available experimental observations. One animal in the saline-control group died during postoperative follow-up before the scheduled week-4 tissue collection. Limited abdominal examination showed non-clotted blood within the pelvic cavity, suggesting postoperative intrapelvic bleeding as the likely cause, although this could not be definitively confirmed in the absence of formal necropsy. Neither animal was replaced or contributed evaluable tissue data. No additional animal or animal-level data exclusions occurred during the study. The remaining 52 animals completed the scheduled follow-up.

No obvious mesh exposure or erosion was observed among the surviving animals. The evaluable sample sizes for H&E staining and quantitative real-time polymerase chain reaction were 6, 5, and 6 in the saline-control, HUCMSC-sEV, and HUCMSC groups at week 1; 5, 6, and 6 at week 4; and 6 per group at week 12. Four additional week-4 samples—two from the saline-control group and two from the HUCMSC group—were unavailable for picrosirius red analysis because of unsuccessful section preparation, leaving 48 evaluable animals. The corresponding week-4 sample sizes were 3, 6, and 4. Missing data were not imputed (Figure 1 and Supplementary Table S7).

### Effects of HUCMSCs and HUCMSC-sEVs on histological responses after mesh implantation

At week 1, inflammatory cell infiltration differed among groups after false discovery rate adjustment (*q* = 0.040), with median scores of 4 [4–4], 3 [3–3], and 3 [3–3] in the saline-control, HUCMSC-sEV, and HUCMSC groups, respectively. Scores were lower in the HUCMSC-sEV group than in the saline-control group (Holm-adjusted *P* = 0.010), whereas the HUCMSC-versus-control comparison was not significant (Holm-adjusted *P* = 0.071).

At week 4, fibrous encapsulation also differed among groups (*q* = 0.040). Median scores were 3 [3–3] in the saline-control group and 2 [2–2] in both intervention groups. Scores were lower in the HUCMSC-sEV and HUCMSC groups than in the saline-control group (Holm-adjusted *P* = 0.017 and 0.007, respectively), with no difference between the intervention groups (Holm-adjusted *P* = 0.642).

The week-12 omnibus comparison for fibrous encapsulation remained significant (*q* = 0.040), with median scores of 3 (2.25–3), 2 (1.25–2), and 2 (2–2), respectively. Both intervention groups had lower scores than the saline-control group in Dunn’s tests (Holm-adjusted *P* = 0.019 and 0.043), but neither comparison remained significant in the exact permutation analysis (Holm-adjusted *P* = 0.097 for both). These week-12 findings should therefore be interpreted cautiously. No other histological outcome–time-point combination remained significant after false discovery rate adjustment (Figure 2, Supplementary Figure S1; Supplementary Tables S1, S2).

**FIGURE 2:**
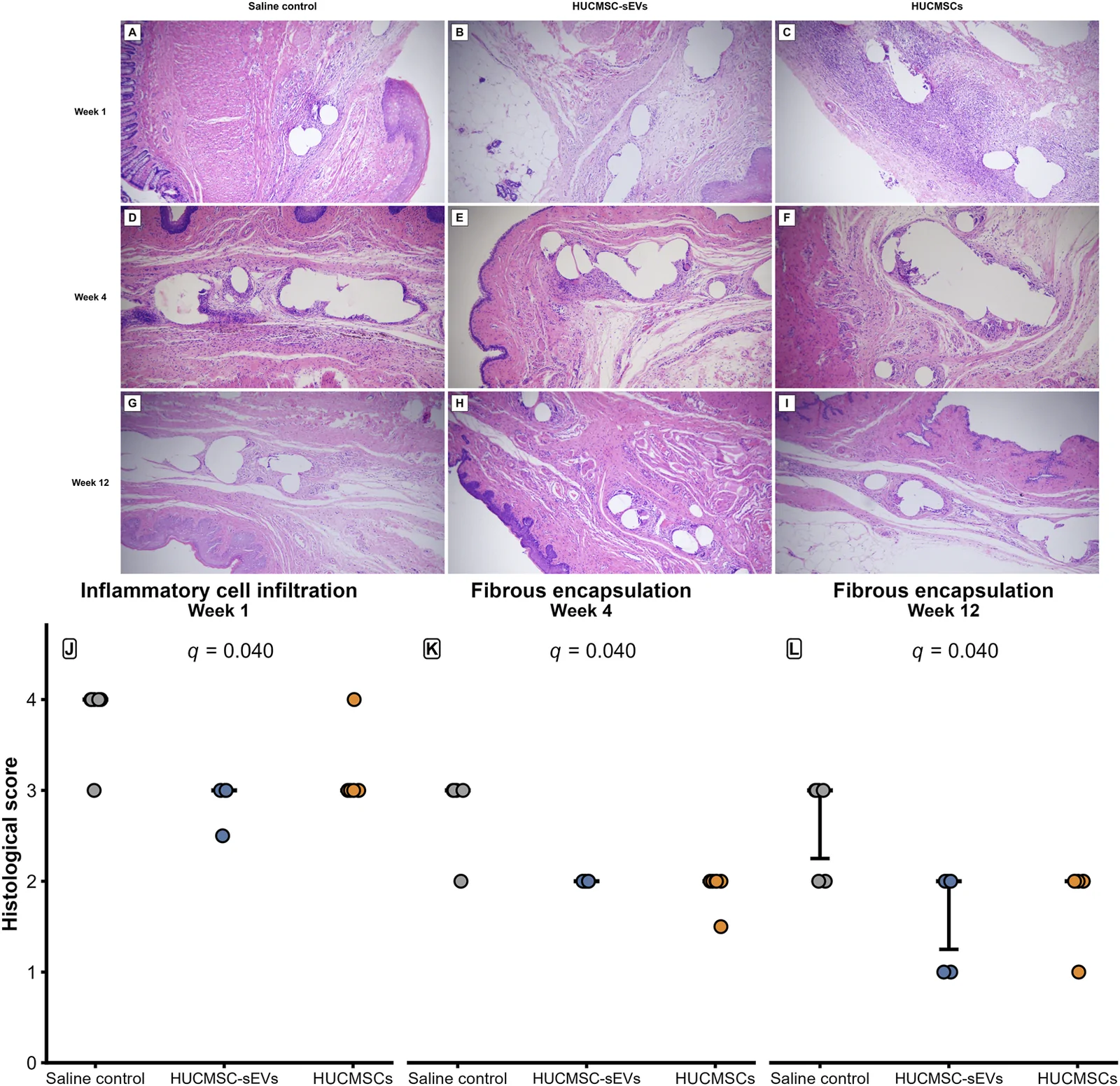
Representative H&E-stained sections and core histological outcomes after mesh implantation. **(A–I)** Representative hematoxylin and eosin (H&E)-stained sections from the saline-control group, HUCMSC-derived small extracellular vesicle (HUCMSC-sEV) group, and human umbilical cord mesenchymal stromal cell (HUCMSC) group at weeks 1, 4, and 12 after mesh implantation. All representative images are shown at the same magnification and are provided for qualitative histological comparison. **(J)** Inflammatory cell infiltration at week 1. **(K)** Fibrous encapsulation at week 4. **(L)** Fibrous encapsulation at week 12. Inflammatory cell infiltration, neovascularization, and fibroblast proliferation were independently evaluated by two observers using a 0–4 ordinal semiquantitative scale, with higher scores indicating a greater degree of the corresponding histological feature. The fibrous encapsulation score was operationally based on semiquantitative assessment of fibroblast proliferation. The arithmetic mean of the two observer scores was used as the animal-level value. Each point represents one animal; horizontal lines and error bars indicate the median and interquartile range. Sample sizes for the saline-control group, HUCMSC-sEV group, and HUCMSC group were 6, 5, and 6 at week 1; 5, 6, and 6 at week 4; and 6, 6, and 6 at week 12, respectively. Overall group differences were assessed using the Kruskal–Wallis test. The nine prespecified outcome–time point comparisons were adjusted using the Benjamini–Hochberg false discovery rate (FDR) procedure. The displayed q values represent FDR-adjusted P values. **(J–L)** show the three outcome–time point combinations that remained significant after FDR adjustment. Pairwise Dunn tests with Holm adjustment are reported in Supplementary Table S2. Complete histological results are presented in Supplementary Figure S1 and Supplementary Tables S1–S3.

Interobserver agreement was high, with linearly weighted Cohen’s *κ* coefficients of 0.898, 0.871, and 0.977 for inflammatory cell infiltration, neovascularization, and fibrous encapsulation, respectively. All paired scores differed by no more than one point (Supplementary Table S3).

### Effects of HUCMSCs and HUCMSC-sEVs on collagen composition

No between-group difference in the COL I-positive area fraction, COL III-positive area fraction, or COL I/COL III ratio was detected at week 1 (Figure 3 and Supplementary Figure S2).

**FIGURE 3:**
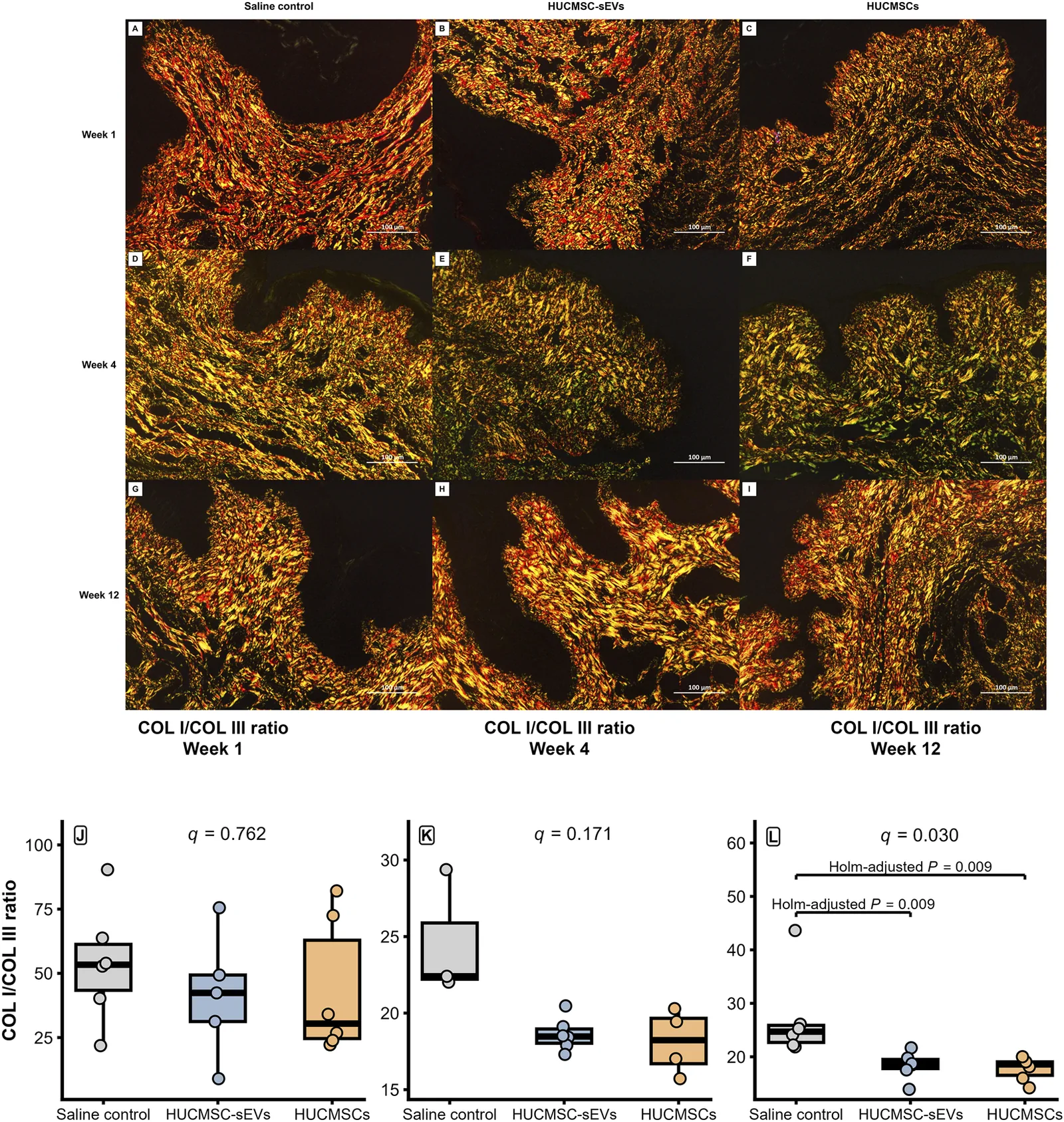
Picrosirius red staining and animal-level COL I/COL III ratios after mesh implantation. **(A–I)** Representative picrosirius red-stained sections viewed under polarized light from the saline-control group, HUCMSC-derived small extracellular vesicle (HUCMSC-sEV) group, and human umbilical cord mesenchymal stromal cell (HUCMSC) group at weeks 1, 4, and 12 after mesh implantation. Scale bars, 100 μm. **(J–L)** Animal-level COL I/COL III ratios at weeks 1, 4, and 12, respectively. Six microscopic fields were quantified for each animal. The primary animal-level COL I/COL III ratio was calculated as the arithmetic mean of the six field-level ratios; the animal, rather than the individual microscopic field, was treated as the experimental unit. Each point represents one animal. Boxes indicate the median and interquartile range, and whiskers extend to the most extreme observations within 1.5 times the interquartile range. Sample sizes for the saline-control group, HUCMSC-sEV group, and HUCMSC group were 6, 5, and 6 at week 1; 3, 6, and 4 at week 4; and 6, 6, and 6 at week 12, respectively. Overall group differences were evaluated using the Kruskal–Wallis test. The nine prespecified outcome–time point comparisons were adjusted using the Benjamini–Hochberg FDR procedure, and the displayed *q* values represent Benjamini–Hochberg FDR-adjusted *P* values. When the overall comparison remained significant after FDR adjustment, pairwise Dunn tests with Holm adjustment were performed; Holm-adjusted *P* values are shown above the relevant comparisons. Complete COL I-positive area fractions, COL III-positive area fractions, ratio analyses, and sensitivity analyses are provided in Supplementary Figure S2 and Supplementary Tables S4–S6.

At week 4, median COL I/COL III ratios were 22.40 [22.22–25.89], 18.47 [18.03–18.96], and 18.23 [16.69–19.66] in the saline-control, HUCMSC-sEV, and HUCMSC groups, respectively. Although both intervention groups showed lower ratios, the overall comparison did not remain significant after false discovery rate adjustment (unadjusted *P* = 0.038, *q* = 0.171) and was considered descriptive.

At week 12, the COL I/COL III ratio differed among groups (*q* = 0.030). Median ratios were 24.70 [22.68–25.88], 18.61 [17.79–19.50], and 18.58 [16.54–19.05], respectively. Ratios were lower in both intervention groups than in the saline-control group (Holm-adjusted *P* = 0.009 for both), with no difference between the HUCMSC-sEV and HUCMSC groups (Holm-adjusted *P* = 0.914). Both intervention-versus-control findings remained significant in the exact permutation analysis (Holm-adjusted *P* = 0.004 for both). The week-12 COL III-positive area fraction did not differ significantly among groups after false discovery rate adjustment (*q* = 0.284), and no adjusted group difference in the COL I-positive area fraction was detected at any time point (Supplementary Tables S4–S6).

### Early changes in the expression of inflammation-related genes

At week 1, IL-10 expression was lower in both the HUCMSC-sEV and HUCMSC groups than in the saline-control group, with relative expression values of 0.055 and 0.084 and corresponding *q* values of 0.010 and 0.021. IL-12 expression was 0.010 and 0.017 (*q* < 0.001 and *q* = 0.015), while TNF-α expression was 0.074 and 0.066 (*q* = 0.006 and *q* = 0.005), respectively.

TGF-β expression was lower in the HUCMSC-sEV group (relative expression, 0.010; *q* < 0.001), and this finding was supported by the nonparametric sensitivity analysis. The corresponding HUCMSC-group result was significant in the parametric analysis (relative expression, 0.022; *q* = 0.020) but not in the nonparametric analysis (*q* = 0.051), indicating limited robustness. No intervention-versus-control comparison for COL1, COL3, MMP2, MMP9, TIMP1, or VEGF remained significant after correction. Exploratory comparisons between the HUCMSC-sEV and HUCMSC groups were also nonsignificant after false discovery rate adjustment (Figure 4, Supplementary Figure S3; Supplementary Table S9A–E).

**FIGURE 4:**
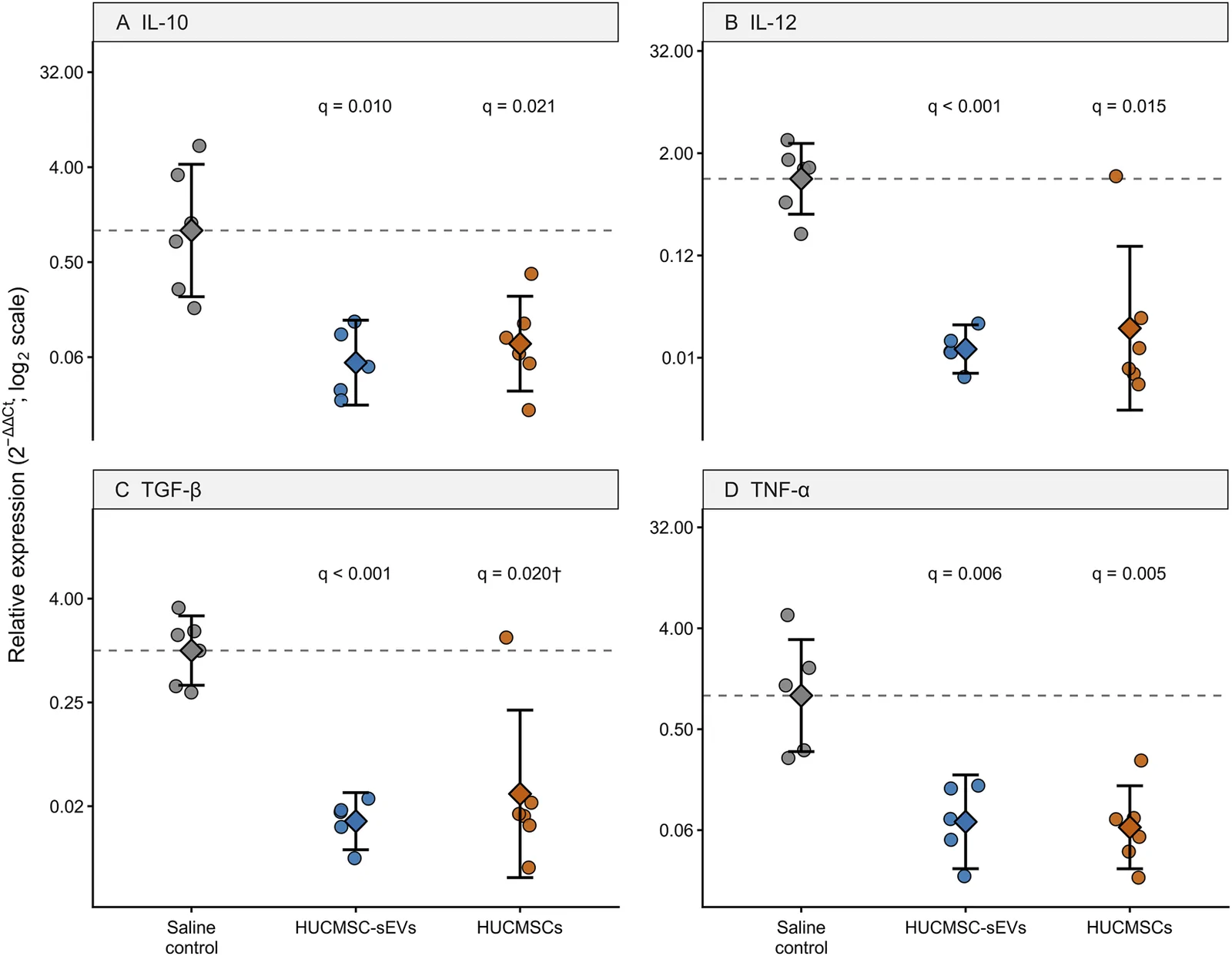
Early cytokine-related mRNA expression at week 1 after mesh implantation. Relative mRNA expression of **(A)** interleukin-10 (IL-10), **(B)** interleukin-12 (IL-12), **(C)** transforming growth factor-β (TGF-β), and **(D)** tumor necrosis factor-α (TNF-α) in the saline-control group, HUCMSC-derived small extracellular vesicle (HUCMSC-sEV) group, and human umbilical cord mesenchymal stromal cell (HUCMSC) group. Each sample–gene combination was assayed in three technical replicate wells, and the mean cycle threshold value after quality control was used for analysis. Relative expression was calculated using the 2^^−ΔΔCt^ method, normalized to β-actin, and calibrated to the mean ΔCt of the saline-control group at the same time point. Individual circles represent animals, diamonds indicate group geometric means, and error bars indicate 95% confidence intervals obtained by back-transforming the corresponding intervals from the ΔCt scale. The horizontal dashed line represents a relative expression value of 1. Sample sizes for the saline-control group, HUCMSC-sEV group, and HUCMSC group were 6, 5, and 6, respectively. Primary pairwise comparisons were performed on ΔCt values using Welch’s t-test. Benjamini–Hochberg FDR adjustment was applied across the 60 prespecified intervention-versus-control comparisons, and the displayed q values correspond to comparisons of each intervention group with the saline-control group. Only comparisons with q < 0.05 are annotated. †The parametric comparison remained significant after FDR adjustment, whereas the corresponding exact Mann–Whitney sensitivity analysis did not meet the FDR significance threshold (q = 0.051). Complete week-1 qPCR results are provided in Supplementary Figure S3 and Supplementary Table S9A–E.

### Changes in the expression of extracellular matrix remodeling-related genes at intermediate and later time points

At week 4, MMP9 mRNA expression was higher in the HUCMSC-sEV and HUCMSC groups than in the saline-control group, with relative expression values of 4.95 and 5.29, respectively (*q* < 0.001 for both). MMP2 mRNA expression was also higher in both groups, with values of 12.73 and 13.83 (*q* < 0.001 for both). These findings were supported by the nonparametric sensitivity analyses. VEGF expression in the HUCMSC group was higher in the parametric analysis (relative expression, 2.84; *q* = 0.042), but this finding was not supported by the nonparametric analysis and was not considered primary. No other week-4 intervention-versus-control comparison remained significant after false discovery rate adjustment.

At week 12, MMP9 mRNA expression was 11.05 and 9.85 in the HUCMSC-sEV and HUCMSC groups, respectively, while MMP2 mRNA expression was 4.36 and 3.79. Both genes were more highly expressed in the intervention groups than in the saline-control group (*q* < 0.001 for all comparisons). COL3 expression was higher in the HUCMSC group (relative expression, 3.84; *q* = 0.006), with support from the nonparametric sensitivity analysis, but did not differ between the HUCMSC-sEV and saline-control groups. No intervention-versus-control comparison for COL1, IL-10, IL-12, TGF-β, VEGF, TIMP1, or TNF-α remained significant at week 12. Exploratory comparisons between the two intervention groups at weeks 4 and 12 were also nonsignificant after adjustment (Figure 5, Supplementary Figures S4–S6; Supplementary Table S9A–E).

**FIGURE 5:**
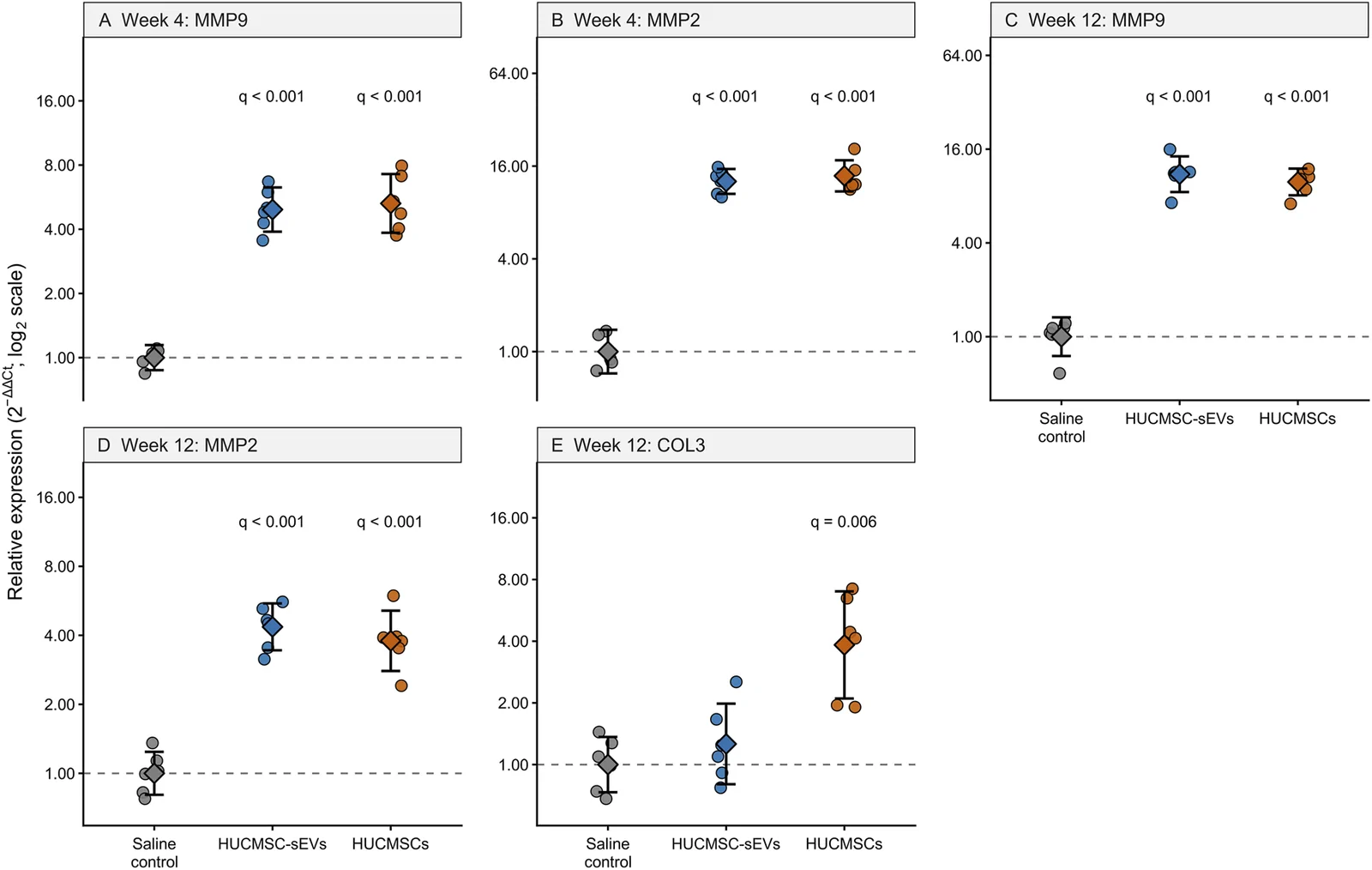
Extracellular matrix remodeling-related mRNA expression at weeks 4 and 12 after mesh implantation. Relative mRNA expression of **(A)** matrix metalloproteinase-9 (MMP9) at week 4, **(B)** matrix metalloproteinase-2 (MMP2) at week 4, **(C)** MMP9 at week 12, **(D)** MMP2 at week 12, and **(E)** type III collagen (COL3) at week 12 in the saline-control group, HUCMSC-derived small extracellular vesicle (HUCMSC-sEV) group, and human umbilical cord mesenchymal stromal cell (HUCMSC) group. Each sample–gene combination was assayed in three technical replicate wells, and the mean cycle threshold value after quality control was used for analysis. Relative expression was calculated using the 2^^−ΔΔCt^ method, normalized to β-actin, and calibrated to the mean ΔCt of the saline-control group at the corresponding time point. Individual circles represent animals, diamonds indicate group geometric means, and error bars indicate 95% confidence intervals obtained by back-transforming the corresponding intervals from the ΔCt scale. The horizontal dashed line represents a relative expression value of 1. Sample sizes for the saline-control group, HUCMSC-sEV group, and HUCMSC group were 5, 6, and 6 at week 4 and 6, 6, and 6 at week 12, respectively. Primary pairwise comparisons were performed on ΔCt values using Welch’s t-test. Benjamini–Hochberg FDR adjustment was applied across the 60 prespecified intervention-versus-control comparisons, and the displayed q values correspond to comparisons of each intervention group with the saline-control group. Only comparisons with q < 0.05 are annotated. Complete week-4 and week-12 qPCR profiles and nonparametric sensitivity analyses are provided in Supplementary Figures S4–S6 and Supplementary Table S9A–E.

## Discussion

### Main findings

In this rat model of posterior vaginal wall mesh implantation, local administration of HUCMSCs and HUCMSC-sEVs was associated with different host-response features at the three independently assessed terminal time points. At week 1, inflammatory cell infiltration was lower in the HUCMSC-sEV group, and IL-10, IL-12, and TNF-α mRNA expression was lower in both intervention groups. At week 4, both interventions were associated with lower fibrous encapsulation and higher MMP2 and MMP9 mRNA expression. At week 12, both groups showed a lower COL I/COL III ratio and higher MMP2 and MMP9 mRNA expression than the saline-control group, while COL3 expression was higher in the HUCMSC group. Taken together, these findings describe distinct host-response patterns at the three independently assessed time points rather than longitudinal changes within the same animals.

### Early inflammatory and cytokine-transcript changes

Polypropylene mesh rapidly induces inflammatory cell recruitment and local cytokine release. Although a controlled inflammatory response is necessary for tissue repair, excessive or prolonged inflammation can promote fibrosis, tissue degeneration, and mesh-related complications (Nolfi et al., 2016; Mangir et al., 2020; Deffieux et al., 2024). At week 1, IL-10, IL-12, and TNF-α mRNA expression was lower in both intervention groups than in the saline-control group, while TGF-β mRNA expression was also lower in the HUCMSC-sEV group. These concurrent changes involved both proinflammatory and regulatory cytokine transcripts and should therefore not be interpreted as a conventional anti-inflammatory cytokine profile. Rather, they indicate a broader alteration in local cytokine-transcript patterns under the tested conditions. Inflammatory-cell infiltration was also lower in the HUCMSC-sEV group at week 1, indicating a difference in early cellular infiltration. However, this histological finding does not establish the underlying immune mechanism or demonstrate functional improvement of the inflammatory response. Because only a limited panel of cytokine transcripts was assessed, the present data cannot distinguish among altered immune-cell recruitment, cellular composition, transcriptional activity, or the timing of the host response, and they are insufficient to define a specific immunological phenotype.

The histological response observed in the HUCMSC-sEV group at the week-1 assessment is consistent with a potential role for HUCMSC-derived paracrine signaling after implantation. Extracellular vesicles can carry biologically active proteins, lipids, messenger RNAs, and microRNAs capable of modulating intercellular communication and inflammatory signaling (Beninson and Fleshner, 2014; Suh et al., 2021; Wei et al., 2023). These properties provide a biologically plausible basis for the altered early host-response patterns observed in the present model.

### Fibrous encapsulation and extracellular matrix remodeling

Fibrous encapsulation is a central component of the host response to implanted biomaterials. Moderate fibrous tissue formation contributes to mesh fixation, whereas excessive encapsulation may increase tissue stiffness, impair integration, and promote contraction (Abhari et al., 2021; Verhorstert et al., 2021; Ács et al., 2024). At week 4, fibrous encapsulation was lower in both the HUCMSC-sEV and HUCMSC groups than in the saline-control group. This finding indicates that both interventions were associated with lower fibrous encapsulation at the week-4 assessment.

At week 12, the COL I/COL III ratio was lower in both intervention groups. Type I collagen contributes mainly to tensile strength and stiffness, whereas type III collagen is associated with a more compliant and actively remodeling matrix (López De Padilla et al., 2021; Gardeazabal and Izeta, 2024). However, neither the COL I-positive area fraction nor the COL III-positive area fraction differed significantly among groups after adjustment. The ratio should therefore be interpreted as a relative measure of collagen composition rather than as direct evidence of reduced fibrosis, improved tissue quality, or enhanced functional integration.

COL3 mRNA expression was increased at week 12 in the HUCMSC group but not in the HUCMSC-sEV group. This difference indicates that the molecular responses of the two interventions were not identical, despite the lower COL I/COL III ratio observed in both groups. Accordingly, the ratio findings should not be taken to imply that HUCMSCs and HUCMSC-sEVs produced the same collagen-remodeling response or acted through the same underlying mechanisms. COL3 transcript abundance and picrosirius red-positive area fractions reflect different biological levels and may not change in parallel. COL3 protein abundance, collagen synthesis and degradation rates, collagen organization, and biomechanical properties were not measured. The present findings should therefore be interpreted as changes in collagen-related measures rather than as direct evidence of improved tissue integration.

Higher MMP2 and MMP9 mRNA expression was observed in both intervention groups at both weeks 4 and 12. MMP2 and MMP9 are involved in the degradation and reorganization of extracellular matrix components (Lu et al., 2015; Okura et al., 2025). In the present study, increased MMP2 and MMP9 transcript levels occurred alongside reduced fibrous encapsulation at week 4 and a lower COL I/COL III ratio at week 12. These parallel findings are compatible with a remodeling-related tissue response (Ruiz-Zapata et al., 2016; Sherrill et al., 2021) but should not be interpreted as direct evidence of enhanced or beneficial extracellular matrix turnover. Because MMP protein abundance and enzymatic activity were not measured, and the endogenous inhibitor profile was incompletely characterized, the functional consequences of the observed transcriptional changes cannot be determined from the present data.

### Comparison between HUCMSCs and HUCMSC-sEVs

The HUCMSC-sEV and HUCMSC groups showed partially overlapping response patterns across the assessed histological and molecular outcomes. At week 1, both groups showed lower IL-10, IL-12, and TNF-α mRNA expression than the saline-control group. Both interventions were also associated with reduced fibrous encapsulation at week 4, lower COL I/COL III ratios at week 12, and higher MMP2 and MMP9 mRNA expression at weeks 4 and 12. However, COL3 mRNA expression increased only in the HUCMSC group, indicating that the later collagen-related molecular responses of the two interventions were not identical. Taken together, these findings indicate partially overlapping, but not identical, response patterns between HUCMSC-sEVs and HUCMSCs across the assessed histological and molecular outcomes.

HUCMSCs can influence tissue responses through multiple mechanisms, including cell-dependent processes and paracrine signaling. Extracellular vesicles represent one potential component of this paracrine communication (Bussche et al., 2015; Chen et al., 2025). The partially overlapping response patterns observed in the HUCMSC and HUCMSC-sEV groups are compatible with the hypothesis that HUCMSC-sEVs may contribute to some HUCMSC-associated paracrine effects in this model. However, these parallel responses do not establish that sEVs are responsible for the effects of HUCMSCs or quantify their relative contribution. Other soluble factors and cell-dependent mechanisms may also be involved. Because sEV-mediated signaling was not selectively perturbed in the present study, causality cannot be inferred from these findings.

HUCMSC-sEVs may also offer practical advantages as a cell-free approach, including greater amenability to storage and dose standardization and avoidance of issues related to living-cell viability, differentiation, and prolonged tissue retention. The present findings support further evaluation of HUCMSC-sEVs as a cell-free approach to modulating mesh-associated host responses.

### Strengths and limitations

This study integrated histological assessment, collagen morphometry, and gene-expression analysis across three independent terminal time points, providing observations of host-response features at early, intermediate, and later assessment time points. Treating the individual animal as the experimental unit avoided pseudoreplication from microscopic fields and technical replicate wells. The use of false discovery rate correction, nonparametric sensitivity analyses, and interobserver agreement assessment further strengthened the statistical and methodological rigor of the study.

Several limitations should be considered. Sample sizes were small, limiting statistical power, particularly for the week-4 picrosirius red analysis, in which only three saline-control animals were evaluable; these findings were therefore interpreted descriptively after FDR correction. Two animals died before scheduled assessment, and the absence of formal necropsy limited causal attribution and safety interpretation. The individual COL I- and COL III-positive area fractions may also be influenced by variation in tissue occupancy within microscopic fields, although this denominator issue does not apply to the within-field COL I/COL III ratio. In addition, the week-12 fibrous encapsulation findings were not confirmed by the exact permutation sensitivity analysis.

The ovariectomized rat model also has limitations in clinical generalizability. Estrogen deficiency was not directly confirmed by serum estradiol or vaginal histology, no sham or no-mesh control was included, and biomechanical properties were not assessed. Accordingly, comparisons with the saline-treated mesh control cannot establish normalization toward non-implanted vaginal tissue. The model differs from clinical pelvic floor reconstruction in anatomy, mesh dimensions, and mechanical loading, and the 12-week follow-up was insufficient to assess long-term mesh exposure, erosion, contraction, pain, or functional integration. Findings obtained with a single titanium-coated polypropylene mesh (TiLOOP® Mesh light) should not be extrapolated to other mesh materials or pelvic floor implants.

Molecular analyses were limited mainly to transcript-level outcomes. Protein abundance and MMP enzymatic activity were not measured, and only TIMP1 was assessed; therefore, the net MMP–TIMP balance and actual extracellular matrix-degrading activity cannot be determined. Cytokine protein levels, immune-cell subsets, macrophage polarization, and spatial or temporal cytokine distributions were also not assessed, precluding definition of a specific immunological phenotype or generalized suppression of inflammation. For the commercially obtained HUCMSCs, complete archived MSC characterization, including quantitative flow cytometry and chondrogenic differentiation, as well as donor-level procurement, ethics, and consent documentation, was unavailable.

For the sEV preparation, specific endosomal biogenesis and batch-to-batch biological reproducibility were not established. Although cell viability remained above 92% after 48 h of serum-free conditioning, effects of serum deprivation on HUCMSC physiology or sEV production and composition cannot be excluded. sEV release or activity was not selectively perturbed, so a causal contribution of sEVs to HUCMSC-associated effects cannot be determined. HUCMSCs and HUCMSC-sEVs were administered using non-equivalent dosing units with no validated conversion factor, only one dose of each intervention was tested, and local distribution and retention were not tracked. Direct between-intervention comparisons were exploratory and do not establish equivalent efficacy or interchangeability.

Future studies should include larger samples, prospectively defined independent sEV batches with standardized batch-release criteria, dose-ranging designs, protein-level and biomechanical measurements, and longer follow-up.

## Conclusion

Local administration of human umbilical cord mesenchymal stromal cells (HUCMSCs) or HUCMSC-derived small extracellular vesicles (HUCMSC-sEVs) was associated with changes in selected histological and molecular features of the host response to vaginal titanium-coated polypropylene mesh implantation, including early inflammatory-cell infiltration and cytokine-transcript expression, fibrous encapsulation, and later collagen- and matrix-related measures. The two interventions showed partially overlapping, but not identical, response patterns under the tested conditions. These findings support further investigation of HUCMSC-sEVs as a cell-free approach to modulating mesh-associated host responses, but do not establish improved functional mesh integration, therapeutic efficacy, or equivalence with HUCMSCs.

## Data Availability

The original contributions presented in the study are included in the article/Supplementary Material, further inquiries can be directed to the corresponding authors.
