# Supplementary material for: Human umbilical cord mesenchymal stromal cells and their small extracellular vesicles modulate host responses to titanium-coated polypropylene mesh in the vaginal wall of rats
Source: Front Bioeng Biotechnol. 2026 Oct 21;14:1985008. doi: 10.3389/fbioe.2026.1985008 (PMC13731329; doi:10.3389/fbioe.2026.1985008)
Supplement: Supplementary file 1 [file Table1.docx]

Supplementary Figure S1. Complete semiquantitative histological assessment after vaginal mesh implantation. Inflammatory cell infiltration (A, D, and G), neovascularization (B, E, and H), and fibrous encapsulation (C, F, and I) were evaluated at weeks 1, 4, and 12 after vaginal mesh implantation in the saline control, HUCMSC-derived small extracellular vesicle (HUCMSC-sEV), and human umbilical cord mesenchymal stromal cell (HUCMSC) groups. Each outcome was scored independently by two observers using a 0–4 semiquantitative scale, and the mean of the two observer scores was used as the animal-level value. Each circle represents one animal. Black horizontal lines and error bars indicate the median and interquartile range, respectively; when the interquartile range was zero, the error bar collapsed to a horizontal line. Overall differences among the three groups at each time point were assessed using the Kruskal–Wallis test. The nine omnibus *P* values were adjusted using the Benjamini–Hochberg false discovery rate procedure, and the resulting *q* values are displayed in the corresponding panels. Complete pairwise comparisons are provided in Supplementary Table S2. Sample sizes for the saline control, HUCMSC-sEV, and HUCMSC groups were 6, 5, and 6 animals at week 1; 5, 6, and 6 animals at week 4; and 6, 6, and 6 animals at week 12, respectively.

Supplementary Figure S2. Complete quantitative assessment of collagen composition by picrosirius red staining after vaginal mesh implantation. COL I-positive area fraction (A, D, and G), COL III-positive area fraction (B, E, and H), and the COL I/COL III ratio (C, F, and I) were evaluated in the saline control, HUCMSC-derived small extracellular vesicle (HUCMSC-sEV), and human umbilical cord mesenchymal stromal cell (HUCMSC) groups at weeks 1, 4, and 12 after vaginal mesh implantation. For each evaluable tissue section, six microscopic fields were quantified. The animal-level COL I- and COL III-positive area fractions were calculated by averaging the corresponding field-level measurements, whereas the primary animal-level COL I/COL III ratio was calculated as the mean of the six field-level ratios. Each circle represents one animal. Boxes indicate the median and interquartile range, and whiskers extend to the most extreme observations within 1.5 times the interquartile range. Overall differences among the three groups at each time point were assessed using the Kruskal–Wallis test. The nine omnibus *P* values were adjusted using the Benjamini–Hochberg false discovery rate procedure, and the corresponding *q* values are shown in each panel. Complete pairwise comparisons and sensitivity analyses are provided in Supplementary Tables S5 and S6, respectively. The numbers of evaluable animals in the saline control, HUCMSC-sEV, and HUCMSC groups were 6, 5, and 6 at week 1; 3, 6, and 4 at week 4; and 6, 6, and 6 at week 12, respectively.

Supplementary Figure S3. Complete gene-expression profile at week 1 after vaginal mesh implantation. Relative mRNA expression levels of COL1 (A), COL3 (B), IL-10 (C), IL-12 (D), MMP9 (E), TGF-β (F), VEGF (G), TIMP1 (H), TNF-α (I), and MMP2 (J) were assessed in the saline control, HUCMSC-sEV, and HUCMSC groups at week 1 using quantitative real-time polymerase chain reaction (qPCR). Relative expression was calculated using the 2^−ΔΔCt^ method and is presented on a log₂-scaled y-axis. Each circle represents one animal. Diamonds and error bars indicate the group geometric means and corresponding 95% confidence intervals, respectively. The horizontal dashed line indicates a relative expression value of 1. Comparisons between each intervention group and the saline control group were performed using ΔCt values. The displayed *q* values are Benjamini–Hochberg false discovery rate-adjusted *P* values across the prespecified intervention-versus-control comparisons. Only comparisons meeting the prespecified FDR threshold are annotated; complete results are provided in Supplementary Table S9. Sample sizes for the saline control, HUCMSC-sEV, and HUCMSC groups were 6, 5, and 6 animals, respectively. †The corresponding result showed limited robustness in the sensitivity analysis.

Supplementary Figure S4. Complete gene-expression profile at week 4 after vaginal mesh implantation. Relative mRNA expression levels of COL1 (A), COL3 (B), IL-10 (C), IL-12 (D), MMP9 (E), TGF-β (F), VEGF (G), TIMP1 (H), TNF-α (I), and MMP2 (J) were assessed in the saline control, HUCMSC-sEV, and HUCMSC groups at week 4 using quantitative real-time polymerase chain reaction (qPCR). Relative expression was calculated using the 2^−ΔΔCt^ method and is presented on a log₂-scaled y-axis. Each circle represents one animal. Diamonds and error bars indicate the group geometric means and corresponding 95% confidence intervals, respectively. The horizontal dashed line indicates a relative expression value of 1. Comparisons between each intervention group and the saline control group were performed using ΔCt values. The displayed *q* values are Benjamini–Hochberg false discovery rate-adjusted *P* values across the prespecified intervention-versus-control comparisons. Only comparisons meeting the prespecified FDR threshold are annotated; complete results are provided in Supplementary Table S9. Sample sizes for the saline control, HUCMSC-sEV, and HUCMSC groups were 5, 6, and 6 animals, respectively. †The corresponding result showed limited robustness in the sensitivity analysis.

Supplementary Figure S5. Complete gene-expression profile at week 12 after vaginal mesh implantation. Relative mRNA expression levels of COL1 (A), COL3 (B), IL-10 (C), IL-12 (D), MMP9 (E), TGF-β (F), VEGF (G), TIMP1 (H), TNF-α (I), and MMP2 (J) were assessed in the saline control, HUCMSC-sEV, and HUCMSC groups at week 12 using quantitative real-time polymerase chain reaction (qPCR). Relative expression was calculated using the 2^−ΔΔCt^ method and is presented on a log₂-scaled y-axis. Each circle represents one animal. Diamonds and error bars indicate the group geometric means and corresponding 95% confidence intervals, respectively. The horizontal dashed line indicates a relative expression value of 1. Comparisons between each intervention group and the saline control group were performed using ΔCt values. The displayed *q* values are Benjamini–Hochberg false discovery rate-adjusted *P* values across the prespecified intervention-versus-control comparisons. Only comparisons meeting the prespecified FDR threshold are annotated; complete results are provided in Supplementary Table S9. Sample sizes for the saline control, HUCMSC-sEV, and HUCMSC groups were 6 animals per group.

Supplementary Figure S6. Forest plots of gene-expression changes in the intervention groups relative to the saline control group. Points represent estimated log₂ fold changes in mRNA expression for the HUCMSC-sEV and HUCMSC groups relative to the saline control group at weeks 1, 4, and 12. Log₂ fold changes were calculated as −ΔΔCt. Horizontal lines represent the corresponding 95% confidence intervals obtained from Welch comparisons based on ΔCt values. Blue circles indicate HUCMSC-sEVs versus saline control, and orange triangles indicate HUCMSCs versus saline control. The vertical dashed line at zero indicates no difference between groups; positive and negative values indicate higher and lower expression relative to the saline control group, respectively. The confidence intervals were not adjusted for multiple comparisons and therefore should not be interpreted as indicating FDR-adjusted statistical significance. Benjamini–Hochberg-adjusted *q* values for all prespecified intervention-versus-control comparisons are provided in Supplementary Table S9. Sample sizes for the saline control, HUCMSC-sEV, and HUCMSC groups were 6, 5, and 6 animals at week 1; 5, 6, and 6 animals at week 4; and 6, 6, and 6 animals at week 12, respectively.

Supplementary Figure S7. Characterization of human umbilical cord mesenchymal stromal cell-derived small extracellular vesicles (HUCMSC-sEVs). (A) Representative transmission electron microscopy (TEM) images of HUCMSC-sEVs at different imaging scales. The corresponding scale bars are indicated in each micrograph. (B) Representative nanoparticle tracking analysis (NTA) profile showing the particle-size distribution of the HUCMSC-sEV preparation. (C) Western blot analysis of the EV-associated proteins CD9, CD63, HSP70, and TSG101, together with Calnexin as a non-EV/endoplasmic-reticulum-associated marker. Molecular-weight marker lanes are shown alongside the corresponding samples.
